# Effects of pH, dissolved organic matter, and salinity on ibuprofen sorption on sediment

**DOI:** 10.1007/s11356-016-7503-6

**Published:** 2016-08-29

**Authors:** Sanghwa Oh, Won Sik Shin, Hong Tae Kim

**Affiliations:** 1 Institute of Livestock Environmental Management, Daejeon, 34065 Republic of Korea; 2 Department of Environmental Engineering, Kyungpook National University, Daegu, 41566 Republic of Korea; 3 Department of Civil Engineering, Kyungpook National University, Daegu, 41566 Republic of Korea

**Keywords:** Ibuprofen, Sorption, Sediment, pH, Dissolved organic matter, Salinity

## Abstract

Ibuprofen is well known as one of the most frequently detected pharmaceuticals and personal care products (PPCPs) in rivers. However, sorption of ibuprofen onto sediment has not been considered in spite of its high *K*_ow_ (3.5). In this study, the effects of various environmental conditions such as pH (4, 5.3, and 7), the concentrations of dissolved organic matters (0 to 1.0 mM citrate and urea), salinity (0, 10, 20, and 30 part per thousand), and presence of other PPCP (salicylic acid) on ibuprofen sorption were investigated. Linear model mainly fitted the experimental data for analysis. The distribution coefficient (*K*_d_) in the linear model decreased from 6.76 at pH 4 to near zero at pH 7, indicating that neutral form of ibuprofen at pH below p*K*a (5.2) was easily sorbed onto the sediment whereas the sorption of anionic form at pH over p*K*a was not favorable. To investigate the effect of dissolved organic matters (DOMs) on ibuprofen sorption, citrate and urea were used as DOMs. As citrate concentration increased, the *K*_d_ value decreased but urea did not interrupt the ibuprofen sorption. Citrate has three carboxyl functional groups which can attach easily ibuprofen and hinder its sorption onto sediment. Salinity also affected ibuprofen sorption due to decrease of the solubility of ibuprofen as salinity increased. In competitive sorption experiment, the addition of salicylic acid also led to enhance ibuprofen sorption. Conclusively, ibuprofen can be more easily sorbed onto the acidified sediments of river downstream, especially estuaries or near-shore environment with low DOM concentration.

## Introduction

Recently, there has been a growing concern on the chronic toxicity of emerging compounds including pharmaceuticals present in the aquatic environment to nature and human being. However, few data are available for the fate of the pharmaceuticals and personal care products (PPCPs) in the water-sediment system. Ibuprofen is widely used as a pain reliever and exists relatively persistent (half-life = 50 days) in aquatic system so it has been reportedly detected frequently at high concentration of 0.9 μg/L in rivers and estuaries (Hilton et al. 2003; Thomas and Hilton 2004) but ibuprofen sorption onto sediment has not been considered significantly even though it has high octanol-water partition coefficient (log *K*_ow_ of 3.5) (Ternes 1998).

In polluted anaerobic sediments, microbiologically catalyzed sulfate reduction originates in sulfides (acid-volatile sulfide, AVS) and redox potential changes can promote sediment acidification by oxidation of sulfide to sulfate where about 0.3 to 3.0 pH in sediment decreased (Di Nanno et al. 2007). Ionizable organic contaminants (IOCs) like ibuprofen exist in aqueous phase as ionic forms and/or neutral forms. The neutral species are predominant when pH < p*K*a whereas the ionic species prevail when pH > p*K*a (Chen et al. 2004). A lot of previous studies have identified that the hydrophobic sorption reaction is affected by the physicochemical properties of the compounds such as water solubility, octanol/water partition coefficient (*K*_ow_), equilibrium coefficient (*K*_a_), and some environmental conditions (pH, oxidation-reduction potential (ORP), and salinity) (Bowman et al. 2002; Wu and Sun 2010; Tremblay et al. 2005). To date, many of possible situations for ibuprofen sorption are still not fully understood or completely known (Scheytt et al. 2005).

Ibuprofen sorption onto sediment should not be expressed by simple reaction, which means there can be many parameters affecting the sorption. For example, the existence of dissolved organic matters (DOMs) can complicate the ibuprofen sorption mechanisms. Tremblay et al. (2005) reported that DOMs can affect the polycyclic aromatic hydrocarbons (PAHs) sorption because PAHs can also interact with DOMs. Therefore, PPCPs are also probably reacted with DOMs. Temporal and spatial gradients of salinity and DOMs in estuaries or coastal waters are known to affect the solubility of the hydrophobic compounds in water. Many researches also have reported that salinity also can affect sorption of hydrophobic organic compounds on the sediment in estuary or seawater (Oh et al. 2013; Tremblay et al. 2005; Wu and Sun 2010). However, studies on the behavior of PPCPs such as ibuprofen in the complex system with DOM, PPCP, water, and sediment system have almost not been up to now (Pan et al. 2009). Salinity also can affect sorption of hydrophobic organic compounds including ibuprofen on the sediment in estuary; however, there is no available information on the effect of salinity on PPCP sorption.

The aim of this study was to investigate the sorption of ibuprofen onto sediments under a variety of environmental conditions, for instance, pH (4.0, 5.3, and 7.0), DOM concentration, salinity (0 to 30 part per thousand (ppt)), and competitive sorption with other PPCPs (salicylic acid). The pH 5.3 is almost the same as the p*K*a of ibuprofen. Two sorption isotherm models such as linear and Freundlich models fitted the experimental sorption data of the ibuprofen. The parameters of the models such as distribution coefficient and Freundlich coefficient were investigated herein.

## Materials and methods

### Sediment

The uncontaminated sediment was taken from the surface layer (below 5 cm) of a wetland in Changnyung, Republic of Korea (latitude: N 35.4397 and longitude: E 128.4836). The sediment was air-dried, sieved through a 212-μm sieve (no. 70) to remove debris, homogenized, and then stored in sealed containers at room temperature before use.

The pH of the sediment was measured using a pH meter (Orion 290A) at 1/5 (*w*/*v*) of sediment to solution ratio in deionized water. The particle size of the sediment was determined by combining sieving and sedimentation steps (Kettler et al. 2001). The point of zero charge (pzc) was also determined by a batch equilibration technique (Sparks et al. 1996). Brunauer, Emmett, and Teller (BET) surface area was measured by a surface area and pore size analyzer (Quantachrome, USA).

### Ibuprofen and buffer

Ibuprofen sodium (Aldrich Chemical Co., >99 %) and the radio-labelled RS-[carboxyl-^14^C] ibuprofen (American Radiolabeled Chemicals, Inc. (ARC), 55 mCi/mmol, >98 %) were used as a sorbate and a radio-tracer, respectively. The physicochemical properties of ibuprofen are summarized in Table 1. The ^14^C-ibuprofen stock solution was made by means of dilution of the radio-labelled ibuprofen solution with methanol to be about 20,000 cpm/mL. The stock solution of unlabelled ^12^C ibuprofen was prepared with HPLC grade methanol for 10 mg/L of ibuprofen concentration in methanol.

**Table 1:** Physical-chemical properties of the ibuprofen studied

	Ibuprofen	Ibuprofen sodium
Chemical formula	C_13_H_18_O_2_	C_13_H_17_NaO_2_
Structure		
Molecular weight (g/mol)	206.29	228.26
Log *K*_ow_	3.5	3.5
p*K*_a_	4.52	4.52
Solubility in water (mg/L)	21	1.0 × 10^5^

Buffer solutions for pH 4.0 and 7.0 were prepared as background solutions using the ^14^C stock solution, the unlabeled ^12^C stock solution, and electrolyte solution including 1 mM CaCl_2_ · 2H_2_O (Duksan Pure Chemical Co. Korea, 70 %), 0.5 mM MgCl_2_ · 6H_2_O (Duksan Chemical Co. Korea, 98 %), and 0.5 mM Na_2_B_4_O_7_ · 10H_2_O (Sigma Chemical Co., 99.5–105.0 %) before each sorption experiment. The methanol to water ratio was lower than 0.2 % (*v*/*v*) to prevent solvent effect for sorption (Zhang and He 2010). Two hundred milligrams per liter of NaN_3_ (Duksan Pure Chemical Co., 97 %) was used as a bacterial inhibitor.

### Ibuprofen sorption onto sediment

#### Sorption procedure

Ibuprofen sorption experiment was conducted at 25 °C in 40 mL amber vials (Fisher Co.) with Teflon-faced silicone septa. Four grams of the sediment was placed into the vials, and then electrolyte solutions were carefully added into the vials to prepare 0.1, 0.2, 0.4, 0.6, 0.8, and 1.0 mg/L of ibuprofen concentrations with initial radioactivity (^14^C-ibuprofen) of about 2000 cpm/mL as a tracer. After carefully filling the vials with the solution minimizing the headspace, the vials were placed in a rotary shaker and shaken at 200 rpm for 72 h. The solution volume was determined gravimetrically. After mixing, the vials were centrifuged at 2000 rpm for 20 min to separate sediment from solution. One milliliter of supernatant was decanted from the centrifugation and mixed with 10 mL of liquid scintillation cocktail (Ultima Gold, Sigma). The radioactivity of ^14^C-ibuprofen was determined by a liquid scintillation counter (LSC; EG&G Wallac Co., 1220 Quantulus).

The equilibrium concentration of ibuprofen was calculated by Eq. (1) of the ratio of ^14^C-ibuprofen change between initial and final:

1 $$ {C}_e={C}_0\times \frac{C_{(fR)e}}{C_{(iR)0}} $$

where *C*_0_ and *C*_*e*_ are initial and equilibrium concentrations of ibuprofen in solution (mg/L), respectively; C_(*iR*)0_ is the concentration of ^14^C-ibuprofen in initial phase, and C_(*fR*)*e*_ is the concentration of ^14^C-ibuprofen in equilibrium phase (count by CPM or DPM).

The solid phase equilibrium concentrations were calculated by assuming all concentration changes in solution were caused by sorption onto the solid phase. The concentration of ibuprofen sorbed *q*_*e*_ (mg/kg) onto sediment was determined using the following equation (Amin 2008; Bajpai and Bhowmik 2010):

2 $$ {q}_e=\left({C}_0-{C}_e\right)\frac{V}{W} $$

where *C*_0_ and *C*_*e*_ are initial and equilibrium concentrations of ibuprofen in solution (mg/L), respectively; *q*_*e*_ is the equilibrium concentrations of ibuprofen in sediment; *V* is the volume of solution (L) and *W* is the weight of sediment used (kg). All experiments were conducted in duplicate.

#### Effect of pH

If p*K*a values of PPCPs are in the range of experimental pH, PPCPs can be protonated or deprotonated and thus exist as different species, such as cation, zwitterion, neutral, and anion (Pan et al. 2009). In the case of ibuprofen (p*K*a = 5.2), the ibuprofen exists as neutral species at pH < p*K*a, coexists as neutral and anionic species at pH ≈ p*K*a, and exists as anion species at pH > p*K*a.

In this study, we selected three pH conditions (pH 4.0, 7.0, and 5.3) to investigate the difference in the sorption of ibuprofen with neutral, neutral/anionic, and anionic species onto sediment. The pH of the electrolyte solution was adjusted to 4.0 by acetate buffer solution (0.01 M) and to 7.0 by phosphate buffer containing 6.74 g/L K_2_HPO_4_ + 8.34 g/L KH_2_PO_4_. And the initial pH of the solution without pH adjusting is 5.3 that is similar to p*K*a (5.2) of ibuprofen.

#### Effect of dissolved organic matter and salinity

Ibuprofen has been present in most of the rivers so it can be detected in estuaries and ocean water. Estuaries are mixing zones for fresh riverine and coastal ocean water masses featuring temporal and spatial gradients of temperature, salinity, and DOM concentration (Tremblay et al 2005).

To estimate the effect of DOMs on the sorption, ibuprofen sorption experiments were also carried out at 0.01, 0.1, and 1.0 M of sodium citrate or urea concentration. The effect of the amount of added citrate and urea and pH (4 and 5.3) on the ibuprofen sorption was investigated and compared. To estimate the salinity effect on ibuprofen sorption, the sorption experiment was also conducted at 0, 10, 20, and 30 ppt of salinity (0, 1.0, 2.0, and 3.0 g/L NaCl). After sorption, the results from ibuprofen sorption with and without NaCl addition were compared.

#### Competitive sorption

Sorption of a mixture of ibuprofen and salicylic acid was conducted with mixing ibuprofen and salicylic acid of 1:1 weight ratio at the same concentrations such as 0, 0.1, 0.2, 0.4, 0.6, 0.8, and 1.0 mg/L of ibuprofen and salicylic acid. The reaction proceeded for 2 h at 25 °C in an orbital shaker. After competitive sorption, the sorption result was compared to the single sorption results from ibuprofen and salicylic acid. The radio-labelled [^14^C] salicylic acid (American Radiolabeled Chemicals, Inc. (ARC), 55 mCi/mmol) was also used as a radio-tracer.

### Sorption isotherm models

The linear model and the Freundlich model were used to describe the sorption data, because the sorption isotherm patterns were shown almost linear. The linear model is expressed as follows:

3 $$ q={K}_dC $$

where *C* (mg/L) is the aqueous-phase equilibrium concentration, *q* (mg/kg) is the solid-phase equilibrium concentration, and *K*_d_ is the distribution coefficient in the linear model.

The Freundlich model is expressed as follows:

4 $$ q={K}_F{C}^{N_F} $$

where *K*_F_ [$\left(\mathrm{mg}/\mathrm{kg}\right)/{\left(\mathrm{mg}/\mathrm{L}\right)}^{N_F}$] and *N*_F_ (−) are the Freundlich model coefficients.

The sorption model parameters were determined by using a commercial software package, Table Curve 2D® (version 5.0, SPSS, Inc.).

## Results and discussion

### Sediment characteristics

**Fig. 1:**
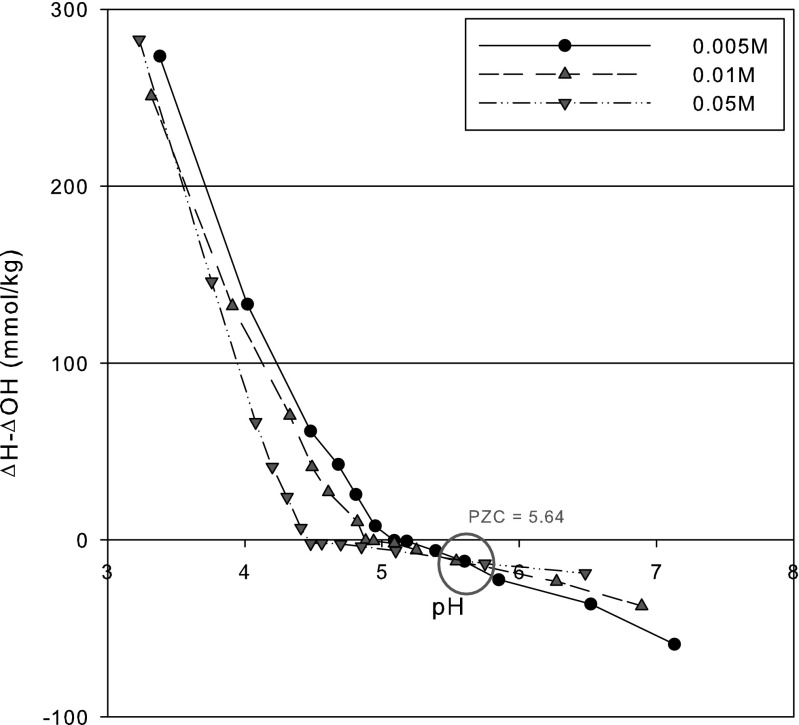
The point of zero charge (pzc) of the sediment used in this study

The pH of the sediment was about 5.49. From analysis of the sediment texture, the fractions of sand, silt, and clay in the sediment were measured at 19.9, 71.2, and 8.9 %, respectively, showing the order of silt > sand > clay. BET surface area of the sediment was 12.278 m^2^/g, and pore volume and radius were 0.056 mL/g and 18.793 A, respectively. Total carbon and organic carbon contents were 0.76 and 0.53 %, respectively. Figure 1 depicts the plotting for estimating the pzc for the sediment, clearly displaying the intersection point at pH 5.64. It indicates that net charge of the sediment is positive when pH is lower than the pzc but negative at higher pH.

### Effect of pH on ibuprofen sorption

**Fig. 2:**
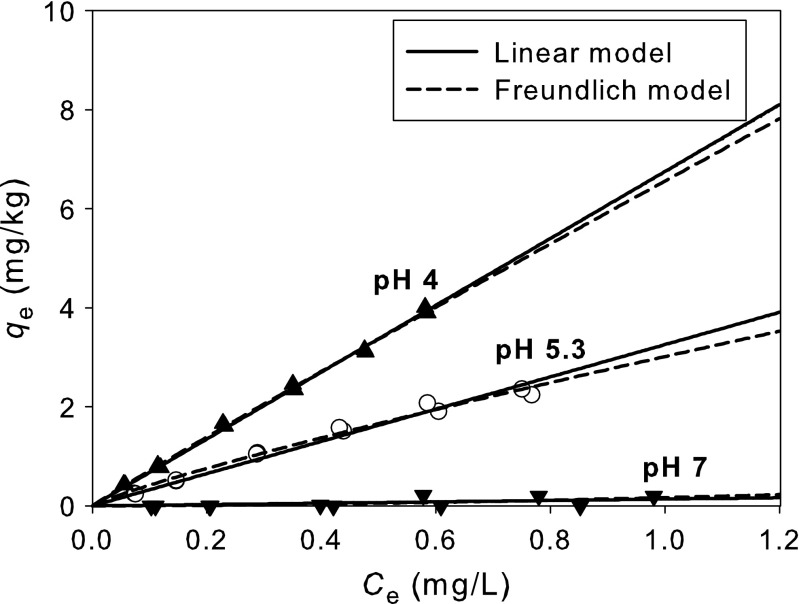
Ibuprofen sorption isotherm onto sediment at pH 4, 5.3, and 7

Pharmaceuticals exist in neutral form (hydrophobic) at pH below p*K*a, but in anionic form (hydrophilic) at pH above p*K*a (Bui and Choi 2009). Ibuprofen exists in the neutral form at pH 4, the neutral/anionic form at p*K*a, and the anionic form at pH 7. In this study, sorption of ibuprofen (p*K*a 5.2) onto sediment was performed at acidic (pH 4), around p*K*a (pH 5.3), and at neutral pH (pH 7). Figure 2 shows the effect of pH on the sorption of ibuprofen onto the sediment at pH 4, 5.3, and 7, and Table 1 presents parameters of the linear and the Freundlich models fitting experimental data.

Figure 2 shows the ibuprofen sorption onto sediment at pH 4, 5.3, and 7. The data are fitted by linear and Freundlich models and the fitted parameters are presented in Table 2. The *R* ^2^ of Freundlich model is almost the same but slightly higher than those of the linear model. Figure 2 shows that the strongly pH-dependent sorption of ibuprofen exhibited almost linear and the ibuprofen sorption was the most favorable at pH 4 whereas it was almost not observed at pH 7. Bui and Choi (2009) also reported that at pH below p*K*a, acidic pharmaceuticals are neutral molecules, interacting with silica surface via non-electrostatic interaction involving hydrogen bonds. In this study, at pH 4 below p*K*a, the neutral ibuprofen was sorbed via non-electrostatic interaction with sediment surface. Although organic and mineral contents are not important in PPCP sorption because of the presence of functional groups in PPCP molecules (Pan et al. 2009), the neutral form is very important in sorption in this study. At pH 7 above p*K*a, ibuprofen was anionic (hydrophilic) and almost not sorbed onto sediment because the sediment was also negatively charged, which led to an electrostatic repulsion with each other (Bui and Choi 2009).

**Table 2:** Model parameters of ibuprofen sorption onto sediment (*n* = 6)

pH	Linear model		Freundlich model		
	*K*_d_	*R* ^2^	*K*_F_	*N*_F_	*R* ^2^
4	6.8 ± 0.1	1.00	6.6 ± 0.1	1.0 ± 0.0	1.00
5.3	3.3 ± 0.1	0.97	3.0 ± 0.1	1.2 ± 0.1	0.99
7	0.1 ± 0.0	0.35	0.2 ± 0.1	0.6 ± 0.4	0.38

Table 2 shows that *K*_d_ values at pH 4, 5.3, and 7 are 6.8, 3.3, and 0.1 L/kg, and *K*_p_ values are 6.6, 3.0, and 0.2, respectively, where *K*_d_ and *K*_p_ values at pH 4 are higher than those at pH 5.3 and 7, and *K*_d_ and *K*_p_ values at pH 5.3 are about in the middle of them at pH 4 and pH 7. This indicates that ibuprofen sorption is strongly affected by its hydrophobicity (neutral form). Bui and Choi (2009) suggested that the pharmaceuticals are sorbed onto soil or sediment by hydrogen bonding between carboxyl groups of pharmaceuticals and silanol groups of soil or sediment particles which is in accordance with this study.

### Effect of dissolved organic matters on ibuprofen sorption

**Fig. 3:**
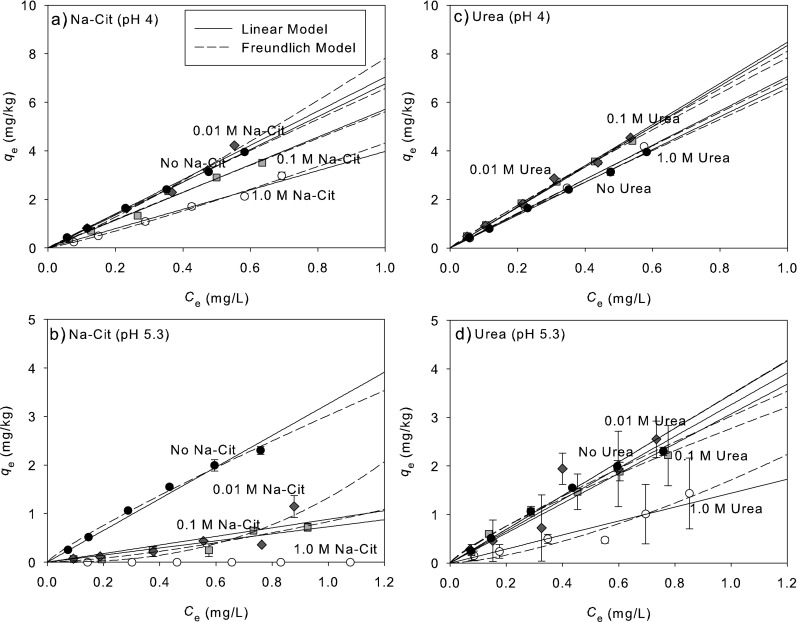
Effect of dissolved organic matters on ibuprofen sorption at pH 4 and 5.3. **a**, **b** Sodium citrate. **c**, **d** Urea. *Black circle*, without Na-Cit or urea; *black diamond*, 0.01 M Na-Cit or urea; *black square*, 0.1 M Na-Cit or urea; *white circle*, 1.0 M Na-Cit or urea

Citrate and urea are considered the representative dissolved organic matters derived from plants and animals, respectively, and have high solubility in water. Gu et al. (2007) observed that PPCPs could bind with DOM in a complicated way because of the presence of functional groups. We conducted sorption isotherm of ibuprofen onto sediment in the presence of citrate and urea with 0 to 1.0 M in solution to estimate the effect of citrate and urea on ibuprofen sorption (Fig. 3). The fitted parameters of linear and Freundlich models are also summarized in Table 3. As seen in Table 3, *K*_F_ values have the same pattern as *K*_d_ values because the fitting results are almost linear.

**Table 3:** Model parameters of ibuprofen sorption onto sediment affected by DOMs (*n* = 6)

DOM	pH	Conc. (M)	Linear model		Freundlich model		
			*K*_d_	*R* ^2^	*K*_F_	*N*_F_	*R* ^2^
Citrate	4	0	6.8 ± 0.1	0.998	6.6 ± 0.2	1.0 ± 0.0	0.999
		0.01	7.0 ± 0.3	0.979	7.8 ± 0.8	0.9 ± 0.1	0.984
		0.1	5.7 ± 0.2	0.982	5.6 ± 0.4	1.0 ± 0.1	0.982
		1	4.0 ± 0.1	0.981	4.3 ± 0.3	0.9 ± 0.1	0.988
	5.3	0	3.3 ± 0.1	0.980	3.0 ± 0.1	1.2 ± 0.1	0.992
		0.01	0.9 ± 0.1	0.672	1.3 ± 0.4	0.4 ± 0.2	0.773
		0.1	0.7 ± 0.1	0.873	0.8 ± 0.1	0.7 ± 0.2	0.914
		1	0	0	0	0	0
Urea	4	0	6.8 ± 0.1	0.998	6.6 ± 0.2	1.0 ± 0.0	0.999
		0.01	8.5 ± 0.2	0.991	8.1 ± 0.5	1.1 ± 0.1	0.993
		0.1	8.3 ± 0.1	0.997	7.8 ± 0.1	1.1 ± 0.0	1.000
		1	7.1 ± 0.2	0.992	6.9 ± 0.4	1.0 ± 0.1	0.992
	5.3	0	3.3 ± 0.1	0.980	3.0 ± 0.1	1.2 ± 0.1	0.992
		0.01	3.5 ± 0.3	0.888	3.5 ± 0.6	1.0 ± 0.3	0.888
		0.1	3.1 ± 0.1	0.966	2.8 ± 0.1	1.2 ± 0.1	0.992
		1	1.4 ± 0.1	0.878	1.7 ± 0.3	0.7 ± 0.2	0.914

As shown in Fig. 3a (sodium citrate), c (urea) at pH 4, the increase in sodium citrate concentration from 0.01 to 1.0 M led to decrease sorption of hydrophobic ibuprofen whereas urea addition did not decrease the ibuprofen sorption even when urea concentration was 1.0 M. This is probably due to little interaction among ibuprofen, urea, and sediment particles. Moreover, from 0.01 to 0.1 M of urea concentration, *K*_d_ values (8.47 and 8.34 L/kg, respectively) of ibuprofen sorption increased a little bit compared to *K*_d_ without urea addition but at 1.0 M urea, *K*_d_ value (7.06 L/kg) was almost the same as the value (6.76 kg/L) without urea addition. This result suggests that ibuprofen sorption can be affected by “salting out effect” (Gu et al. 2007; Oh et al. 2013) if urea was not reacted with ibuprofen.

But as sodium citrate was added, ibuprofen sorption gradually decreased. *K*_d_ values at citrate concentrations of 0, 0.01, 0.1, and 1.0 M were 6.76, 7.04, 5.71, and 3.98 L/kg, respectively (see Table 3). Citrate has three carboxyl groups. Therefore, it is possible that some of ibuprofen was bound to the carboxyl groups. If citrate-ibuprofen complex was formed, the complex also exists anionic and soluble form causing gradual decrease in ibuprofen sorption. At pH 5.3, about half of ibuprofen is also negatively charged. The ibuprofen sorption was remarkably interrupted by citrate addition at only 0.01 M and at high concentration of urea (1.0 M).

### Effect of salinity on ibuprofen sorption

**Fig. 4:**
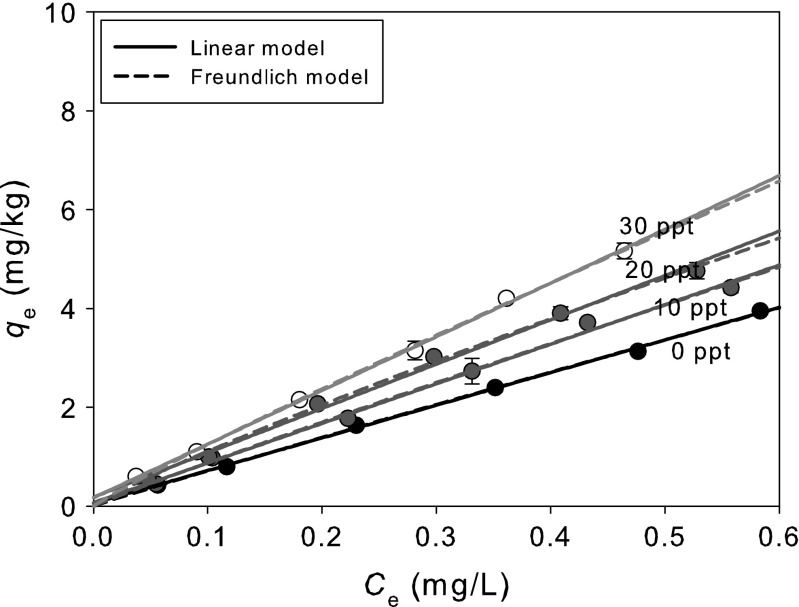
Effect of salinity on ibuprofen sorption

Sorption isotherm of ibuprofen and the relationship between *K*_d_ (and *K*_F_) and salinity are depicted in Fig. 4 and Table 4. Figure 4 illustrates that the sorption behavior of ibuprofen on sediment followed linear isotherm at all different salinities. *K*_d_ for ibuprofen sorption increased with salinity due to “salting out” effect, which is caused by high salinity resulting in high compressive water-water interaction, decreasing water solubility, and less cavitation of water molecule (Turner 2003; Oh et al. 2013). Oh et al. (2013) also reported that log *K*_ow_ of hydrophobic PAHs increased with salinity, indicating that hydrophobic materials may be sorbed easily onto sediment at high salinity. For hydrophobic pollutants, therefore, sorption onto particulate matter is one of the dominant phase transfer processes affecting their movement and fate in the environment (Wu and Gschwend 1986). You et al. (2010) also reported that the increase in salinity results in a decrease in the solubility of organic compounds and thus an increase in sorption coefficient. This experiment was conducted at pH 4 where neutral form of ibuprofen exists, indicating that there is a high possibility of ibuprofen trapped in sediments acidified in estuary or coastal conditions due to high salinity.

**Table 4:** Model parameters for ibuprofen sorption onto sediment at 0, 10, 20, and 30 ppt of salinity (*n* = 6)

pH	Salinity (ppt)	Linear model		Freundlich model		
		*K*_d_	*R* ^2^	*K*_F_	*N*_F_	*R* ^2^
4	0	6.8 ± 0.1	0.998	6.6 ± 0.2	1.0 ± 0.0	0.999
	10	8.2 ± 0.1	0.994	7.9 ± 0.4	1.0 ± 0.0	0.995
	20	9.5 ± 0.2	0.989	8.5 ± 0.3	0.9 ± 0.0	0.997
	30	11.4 ± 0.2	0.995	10.6 ± 0.4	0.1 ± 0.0	0.998

### Bi-solute competitive sorption of ibuprofen and salicylic acid

**Fig. 5:**
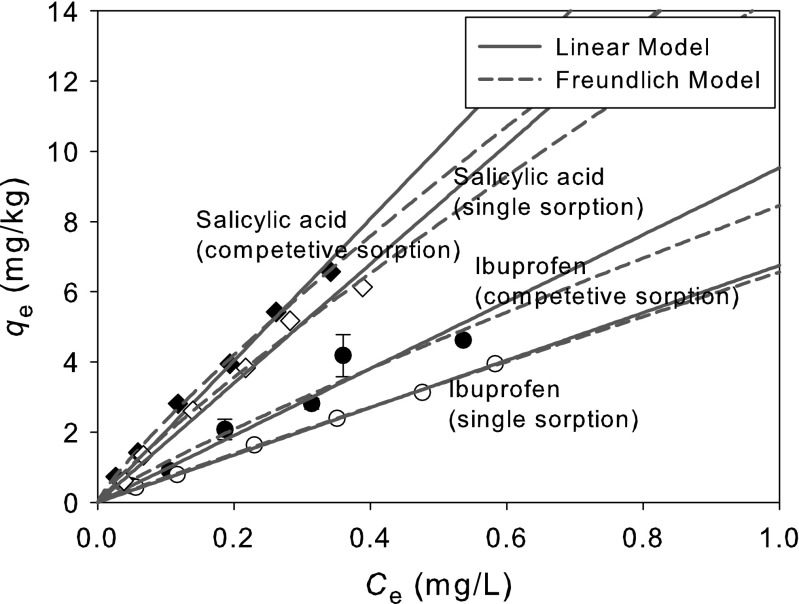
Competitive sorption of ibuprofen and salicylic acid

Bi-solute competitive sorption experiments for ibuprofen and salicylic acid were performed. As expected, when the two pharmaceuticals compete for sorption at the same site of sediment, the sorption of each ibuprofen and salicylic acid was less than that in single-solute system. Single- and bi-solute sorption isotherm is depicted in Fig. 5, and the sorption parameter of the experimental data fitted to linear and Freundlich models are summarized in Table 5.

**Table 5:** Parameters fitted from linear and Freundlich models for single- and bi-solute sorption of ibuprofen and salicylic acid onto sediment at pH 4 (*n* = 6)

PPCP	Mode	Linear model		Freundlich model		
		*K*_d_	*R* ^2^	*K*_F_	*N*_F_	*R* ^2^
Ibuprofen	Single	6.8 ± 0.1	0.998	6.6 ± 0.2	1.0 ± 0.0	0.999
	Competitive	9.5 ± 0.6	0.934	8.5 ± 1.3	1.1 ± 0.0	0.997
Salicylic acid	Single	17.0 ± 0.6	0.979	14.5 ± 1.2	1.1 ± 0.1	0.990
	Competitive	20.2 ± 0.6	0.995	16.5 ± 0.7	1.2 ± 0.0	0.997

In comparison with single-solute sorptions, *K*_d_ values for single- and bi-solute sorption for both solutes are increased from 6.76 to 9.53 L/kg for ibuprofen and from 16.98 to 20.19 L/kg for salicylic acid, respectively. Therefore, the *K*_d_ ratio between bi-solute sorption and single-solute sorption was 1.41 for ibuprofen and 1.19 for salicylic acid, respectively. This indicates that the sorption of ibuprofen and salicylic acid was enhanced as concentrations of them increased. There was a synergistic effect on ibuprofen sorption. Some portion of the ibuprofen in solution also could be sorbed or attached on the particles of salicylic acid which has higher sorption affinity, *K*_d_, than ibuprofen (see Fig. 5). That is probably one of the reasons for the increase in ibuprofen sorption.

## Conclusion

Ibuprofen sorption onto sediment was investigated under several environmental conditions such as pH change, dissolved organic matter, salinity, and competitive sorption to understand the fate of ibuprofen in water-sediment system. Ibuprofen was not sorbed onto sediment at pH 7 whereas it was favorably sorbed at pH 4. Therefore, if sediment is acidified, the ibuprofen changes its form from anionic to neutral that can be easily sorbed onto the sediment. The presence of citrate from 0.01 to 1.0 M interrupted ibuprofen sorption; furthermore, the interruption was enhanced at pH 5.3 as compared to pH 4.0. However, urea did not interrupt but enhanced the ibuprofen sorption. Salinity also enhanced ibuprofen sorption. In bi-solute competitive sorption, addition of salicylic acid could boost the ibuprofen sorption; thus, the *K*_d_ and *K*_F_ also increased. Conclusively, ibuprofen sorption can be found at acidified sediments located around river downstream, estuaries, or near-shore marine environment with low DOM concentration.

## References

[CR1] Amin NL (2008). Removal of reactive dye from aqueous solutions by the adsorption onto activated carbons prepared from sugarcane bagasse pith. Desali.

[CR2] Bajpai SK, Bhowmik M (2010). Adsorption of diclofenac sodium from aqueous solution using polyaniline as a potential sorbent. I. Kinetic studies. J Appl Polym Sci.

[CR3] Bowman JC, Zhou JL, Readman JW (2002). Sediment-water interactions of natural oestrogens under estuarine conditions. Mar Chem.

[CR4] Bui TX, Choi H (2009). Adsorptive removal of selected pharmaceuticals by mesoporous silica SBA-15. J Hazard Mater.

[CR5] Chen H, Zhou W, Zhu K, Zhan H, Jiang M (2004) Sorption of ionizable organic compounds on HDTMA-modified loess soil. Sci Total Environ 326:217–223 10.1016/j.scitotenv.2003.12.011 15142777

[CR6] Di Nanno MP, Curutchet G, Ratto S (2007). Anaerobic sediment potential acidification and metal release risk assessment by chemical characterization and batch resuspension experiments. J Soils Sediments.

[CR7] Gu C, Karthikeyan KG, Sibley SD, Pedersen JA (2007). Complexation of the antibiotic tetracycline with humic acid. Chemosphere.

[CR8] Hilton MJ, Thomas KV, Ashton D (2003) Targeted monitoring programme for pharmaceuticals in the aquatic environment. R&D Technical Report P6-012/06/TR. Environmental Agency, United Kingdom

[CR9] Kettler TA, Doran JW, Gilbert TL (2001). Simplified method for soil particle-size determination to accompany soil-quality analyses. Soil Sci Soc Am J.

[CR10] Oh S, Wang Q, Shin WS, Song DI (2013). Effect of salting out on the desorption-resistance of polycyclic aromatic hydrocarbons (PAHs) in coastal sediment. Chem Eng J.

[CR11] Pan B, Ning P, Xing B (2009). Part V—sorption of pharmaceuticals and personal care products. Environ Sci Pollut Res.

[CR12] Scheytt Y, Mersmann P, Lindstadt R, Heberer T (2005). Determination of sorption coefficients of pharmaceutically active substances carbamazepine, diclofenac, and ibuprofen, in sandy sediments. Chemosphere.

[CR13] Sparks DL, Page AL, Helmke PA, Loeppert RH, Soltanpour PN, Tabatabai MA, Johnston CT, Sumner ME (1996) Methods of soil analysis. Part 3—chemical methods. Soil Science Society of America, Inc. USA

[CR14] Ternes TA (1998). Occurrence of drugs in German sewage treatment plants and rivers. Water Res.

[CR15] Thomas KV, Hilton MJ (2004). The occurrence of selected human pharmaceutical compounds in UK estuaries. Mar Pollut Bull.

[CR16] Tremblay L, Kohl SD, Rice JA, Gagne JP (2005). Effects of temperature, salinity, and dissolved humic substances on the sorption of polycyclic aromatic hydrocarbons to estuarine particles. Mar Chem.

[CR17] Turner A (2003). Salting out of chemicals in estuaries: implications for contaminant partitioning and modelling. Sci Total Environ.

[CR18] Wu S, Gschwend PM (1986). Sorption kinetics of hydrophobic organic compounds to natural sediments and soils. Environ Sci Technol.

[CR19] Wu W, Sun H (2010). Sorption-desorption hysteresis of phenanthrene—effect of nanopores, solute concentration, and salinity. Chemosphere.

[CR20] You C, Jia C, Pan G (2010). Effect of salinity and sediment characteristics on the sorption and desorption of perfluorooctane sulfonate at sediment-water interface. Environ Pollut.

[CR21] Zhang J, He M (2010). Effect of structural variations on sorption and desorption of phenanthrene by sediment organic matter. J Hazard Mater.

